# Dronabinol Is Not a Game Changer in Pediatric Palliative Care: Results from a Retrospective Study

**DOI:** 10.3390/children11091054

**Published:** 2024-08-28

**Authors:** Holger Hauch, Annika Lisakowski, Julia Wager, Boris Zernikow

**Affiliations:** 1 Pediatric Palliative Care Centre, Children’s and Adolescents’ Hospital Datteln, 45711 Datteln, Germany; 2 Department of Children’s Pain Therapy and Pediatric Palliative Care, Faculty of Health, School of Medicine, Witten/Herdecke University, 58455 Witten, Germany; 3 PedScience Research Institute, 45711 Datteln, Germany

**Keywords:** dronabinol, palliative care, tetrahydrocannabinol, restlessness, pain, spasticity, epilepsy, children, pediatric

## Abstract

Background/Objectives: Patients with life-limiting conditions (LLCs) often suffer from restlessness, spasticity, pain, and seizures. Dronabinol (DRB) may have a relieving effect; however, data on the effectiveness of DRB in children with LLCs are limited to outpatients. The aim of this study was to assess the efficacy and safety of DRB. Methods: Retrospective analysis of inpatients. Results: From 2011 to 2021, 1219 patients were admitted. Of these, 63 patients (63.5% male, age: 10.4 (SD = 6.3) years) were treated with DRB; 96.8% had a neurological disease, and 26 patients were started on DRB (group A), while 37 were admitted with existing DRB (group B). The effective doses were 0.21 (SD = 0.11) in group A and 0.48 (SD = 0.5) mg/kg/BW/day in group B (*p* = 0.01). Subjective response rates to DRB in both groups (good/moderate effect) were 9.5%/38.1% for spasticity and 1.6%/25.4% for restlessness. However, no reduction in seizures, restlessness, or demand medication was observed in 24 h protocols when patients started DRB in group A. Three patients experienced severe side effects (e.g., respiratory depression). Other side effects included fatigue (22.2%) and behavioral problems (14.3%). Conclusions: Subjective positive effects could not be confirmed by more objective data. Side effects can be severe. Thus, DRB should be started in a well-monitored setting and only with clear indications.

## 1. Introduction

Cannabis is becoming increasingly prevalent in Europe and the western world [1]. Since the possible reimbursement of costs for medicinal cannabinoids by statutory health insurance companies in Germany in 2017, there has been a rise in prescriptions within the healthcare sector [2]. In 2022, nearly 400,000 prescriptions for medical cannabis were billed to legally insured persons, amounting to a turnover of EUR 198 million [3]. In addition, the use of non-medical cannabis was permitted in Germany in April 2024, meaning that cannabis will likely have a major social impact [4].

The potential benefits of physiological messenger substances in the endocannabinoid system, such as pain-inhibiting, muscle-relaxing, and calming effects, make medical cannabinoids particularly interesting for pediatric palliative care. Children and adolescents with life-limiting conditions (LLC) often have unique and rare diseases [5]. The largest group of these patients suffers from neurological diseases, which frequently have similar accompanying conditions such as severe developmental delays, cerebral palsy, epilepsy, and movement disorders. Restlessness and pain are the most common symptoms [6].

Medicinal cannabinoids can be used in various forms, such as flower components, mixtures of tetrahydrocannabinol (THC) and cannabidiol (CBD), or as a single agent. THC has an antiemetic effect, for instance, compared to domperidone in chemotherapy-induced vomiting [7,8], as well as in autism and other mental disorders [9,10]. CBD, on the other hand, is approved for the treatment of epileptic seizures in Dravet syndrome, Lennox–Gastaut syndrome, and tuberous sclerosis. Previous experience with medicinal cannabinoids in pediatric palliative care is limited [11,12,13]. So far, the available data for children and adolescents with LLCs are restricted to the outpatient sector [12,13,14,15], and the effects on relevant symptoms such as spasticity, restlessness, seizures, and pain have not been sufficiently investigated. Long-term effects of THC in neurologically impaired children, who may have a life expectancy of many years, have not yet been published. Despite the recently published ASCO (American Society of Clinical Oncology) guidelines on treating adults with cancer using medical cannabis/cannabinoids, no standards for treating children with medical cannabinoids have been established at the time of publication [16].

The aim of this study was to investigate the efficacy and safety of DRB treatment. Since 2011, children in the pediatric palliative care unit in Datteln, Germany, have been treated with the semi-synthetic THC preparation dronabinol (DRB). In this retrospective study, we aimed to answer two research questions to draw practical conclusions on the use of DRB in children and adolescents with LLCs. First, we aimed to determine the indications and dosages chosen for DRB therapy. Second, we sought to assess the efficacy and safety of DRB treatment.

## 2. Patients and Methods

A retrospective review of the medical records of all inpatients admitted to the pediatric palliative care unit at the Children’s and Adolescents’ Hospital, Datteln, Witten/Herdecke University, was conducted between 1 January 2011, and 30 September 2023 (see Box 1). Inclusion criteria were the presence of a life-limiting condition (LLC) and initial treatment or first admission after starting outpatient therapy with the semi-synthetic THC preparation dronabinol (DRB). Two different groups were formed: patients starting DRB therapy on the ward (group A) were compared with those admitted with pre-existing DRB therapy (group B).

### 2.1. Patient Data

The patients treated could be reliably identified based on statutory documentation requirements for DRB. Main life-limiting diagnosis, comorbidities, age, gender, weight, age-specific weight z-score [17], and concomitant medications were extracted from patient records. Life-limiting diseases were classified according to the standard scheme of the Together for Short Lives Foundation [18]. Other variables extracted were indications for DRB administration, dosages, and duration of treatment. Vital functions, such as circulatory and oxygen saturation, were monitored in all patients on DRB during sleep or daytime.

Box 1 **Pediatric palliative care unit, Children’s and Adolescents’ Hospital, Datteln, Witten/Herdecke University, Datteln, Germany.**

-Opened as an affiliate of a tertiary care children’s hospital in 2010

-First palliative care unit in Germany and Europe

-A total of 8 beds with 120–170 admissions/year

-Multiprofessional team with bio-psycho-social care approach

-Indications for admission: diagnostics/treatment of burdensome symptoms,

   intervention of acute crisis (e.g., pneumonia, seizures, terminal care),

   optimization of therapy (e.g., home ventilation, antiepileptic/analgesic treatment)

The 24 h protocols prescribed for all patients were included in the evaluation [19]. These protocols documented waking and sleeping phases, seizures, phases of restlessness, and pain.

### 2.2. Effects of Dronabinol

The possible impacts on spasticity, restlessness, seizures, pain, and dystonia, as well as potential side effects, were documented in medical records. For both groups, these effects were classified as “good”, “moderate”, “no effect”, or “worsening”. In group A, observations from different professionals (medical and nursing staff) were treated equally. In group B, DRB effects were reported by parents and documented in medical records. If the assessments from different sources matched, the effect was classified accordingly. If assessments differed, either the median or the less effective level was recorded in the database (e.g., good + no effect = moderate; good + worsening = no effect).

### 2.3. Side Effects

Side effects were recorded from patients’ files. If an adverse event prolonged the hospital stay or was life-threatening, it was classified as severe. In such cases, a review was carried out to check for potential dosage errors, concomitant medication, and the reversibility of the symptoms. Reasons for discontinuation of DRB therapy were also recorded. All other adverse effects with at least a possible causality to the DRB intake were documented according to the WHO-UMC causality assessment system [20].

### 2.4. Follow-Up

Patient records were analyzed to track further contacts with the outpatient clinic, other hospitals, or the affiliated pediatric palliative care team at home to collect data on the longer-term course (duration of therapy and survival) after discharge. The date of last contact was documented, and where possible, information was gathered on whether DRB treatment was continued or stopped. If a case was lost to follow-up, the day of discharge from the pediatric palliative care (PPC) unit was documented as the last contact.

### 2.5. Data Collection and Statistics

Data were collected anonymously and transferred to a database. SPSS 29.0 (IBM Cooperation, NY, USA) and MS Excel (Microsoft Cooperation Seattle, WS, USA) were used for analysis. In addition to descriptive statistical methods, tests for the comparison of variables (Wilcoxon tests, Mann–Whitney U tests, chi-square tests) were carried out. Survival time estimates were calculated using the Kaplan–Meier method. *p*-values were adjusted according to Benjamini and Hochberg [21]. A *p*-value < 0.05 was considered significant. Effect sizes were calculated using Pearson’s method and interpreted according to Cohen’s criteria (0.1 = weak, 0.3 = moderate, 0.5 = high) [22]. Ethical approval was obtained from the Ethics Committee of Witten/Herdecke University (approval code: S-316/2023, approval date: 12 December 2023). At admission, parents were provided with a general consent for data collection and scientific use of data. The study and manuscript preparation followed the STROBE recommendations [23].

## 3. Results

### 3.1. Demographics

A total of 1219 patients were treated in the PPC unit during the study period, with 63 of them (5.2%) receiving DRB. Of these, 26 (41%) patients started DRB on the PPC unit (group A), while the remaining 37 patients (59%) were already on DRB as part of their regular medication upon admission (group B). Groups A and B did not differ in demographic or medical parameters at admission. Therefore, characteristics of the whole sample are presented in Table 1.

A severe underlying neurological condition was present in 96.8% of the patients. The largest subgroup comprised children with hypoxic brain damage (e.g., due to asphyxia or after resuscitation), followed by metabolic diseases and malformations. Considering gender, 63% were male. Common co-morbidities included developmental and movement disorders, as well as epilepsy. Patients with epilepsy were receiving a median of two anticonvulsant medications (range 1–5). Almost a third of the patients were prescribed an opioid (mostly morphine as 0.5% drops via a PEG tube) for musculoskeletal pain, and in nearly half of the cases, additional benzodiazepines or other sedatives were administered.

**Figure 1:**
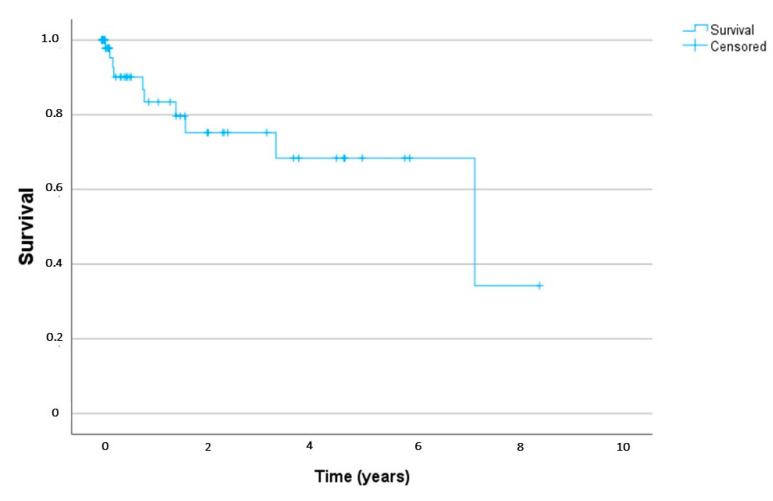
Survival of all study patients (Kaplan–Meier).

Groups A and B did not differ in the indication for treatment with DRB. The main symptoms that led to DRB treatment were restlessness, spasticity, and pain. As can be seen in Figure 1, the 5-year survival rate of all patients analyzed in this study was 68% after admission to the PPC unit.

### 3.2. Treatment

Table 2 details the characteristics of the two groups: DRB-naive patients (group A) and chronic DRB users (group B). Patients in group B started DRB a median of 128 days (range: 14–579 days) before admission to the PPC unit (in the outpatient setting). Upon admission, the average DRB dose for this group was 0.48 mg/kg/bw (SD: 0.5). Information regarding starting doses or dosing steps was not available for group B. In contrast, patients in group A started with an initial dose of DRB of 0.06 mg/kg/bw and required an average of three dosing steps to reach the effective average dose of 0.21 mg/kg/bw. The effective dose in group A was significantly lower than in group B (0.48 mg/kg/bw; *p* = 0.01).

### 3.3. Efficacy

The subjectively documented efficacy rate of DRB (at least moderate response) for both groups (A/B) was 9.5%/38.1% for spasticity (*n* = 42), 1.6%/25.4% for restlessness (*n* = 44), 1.6%/12.7% for pain (*n* = 37), 3.2%/12.7% for seizures (*n* = 11), 0%/6.3% for dystonia (*n* = 9), and 1.6%/7.9% for myoclonus (*n* = 6).

The effects of DRB in group A were investigated in more detail using standardized 24 h protocols and recordings. After starting DRB, the daily mean rate of seizures decreased from 0.7 (SD = 2.7) to 0.5 (SD = 2.0), but this change was not significant (*p* = 0.106). The mean number of episodes of restlessness was 2.3/day (SD = 1.8) before starting DRB and 2.2/day (SD: 1.9) (*p* = 0.160) after starting DRB. There was no reduction in demand medication under DRB treatment. Before starting DRB, 0.15 doses of demand analgesics were administered per day (SD = 0.4), and after starting DRB, this remained stable at 0.15/day (SD = 0.4) (*p* = 0.773). Daily demand rates for sedatives were 0.3 (SD = 0.5) before and 0.2 (SD = 0.4) (*p* = 0.345) after starting DRB.

### 3.4. Side Effects

Three episodes were detected during the observation period after DRB was started. See synopsis in Table 3 and the detailed case descriptions.

### 3.5. Case Descriptions

#### 3.5.1. Case 22

A 13-year-old boy (weight Z-score: −3.44) suffered from an etiologically unclear severe developmental delay with morphologically cortical atrophy and bilateral hippocampal sclerosis. Upon admission, the patient received Levetiracetam, Clobazam, Phenytoin, Valproate, Vigabatrin, Omeprazole, and Melatonin. The EEG consistently showed seizure equivalents. DRB was initiated due to severe restlessness. The initial dose was 0.09 mg/kg/day/bw, which was not increased until day three. On the third day, the patient experienced altered consciousness, episodes of crying, and respiratory insufficiency requiring oxygen therapy. Measurements of antiepileptic medication levels, ultrasound, and lab work provided no explanation for these symptoms. No other medication was changed or added on that day or the two days before. The phase of respiratory insufficiency was not suspicious of a seizure. After discontinuing DRB, the problem resolved completely. Reintroduction of DRB therapy was not attempted.

#### 3.5.2. Case 25

A 1,6-year-old male toddler (weight Z-score: 0.1) was diagnosed with multiple perinatal hemorrhagic brain infarcts and presented with global developmental delay and refractory epilepsy. At the time of the side effect, the patient was given Clobazam, Valproate, Morphine, Esomeprazole, and Domperidone. Due to severe agitation, the boy was given increasing doses of Dronabinol. On day 14, at a DRB dosage of 0.30 mg/kg/day, an acute respiratory insufficiency occurred, requiring non-invasive ventilation. The remaining medication was not changed. After discontinuing DRB, his respiration completely normalized, and the issue did not recur. Reintroduction of DRB therapy was not attempted.

#### 3.5.3. Case 35

A 9,8-year-old boy (weight Z-score: −1.1) was diagnosed with adenyl succinate lyase deficiency (MIM:103050) and admitted due to severe agitation. The patient had profound developmental delay and refractory epilepsy. His medication consisted of Valproate, Lamotrigine, Oxcarbazepine, Morphine, Melperone, Omeprazole, Melatonin, and Rufinamide. On day 12, at a DRB dose of 0.25 mg/kg/bw/day, reduced nighttime respiratory rates were observed at nine breaths per minute. Upon repeated stimulation, the respiratory rate rose but dropped again after discontinuation of inducement. The phase of respiratory insufficiency was not suspicious of a seizure. No other medications were added or changed in the two days prior to this incident. As DRB had no effect on restlessness, it was discontinued.

Other adverse effects with at least a possible causality to DRB intake were fatigue (22.2%), behavioral problems (14.3%), diarrhea (9.5%), constipation (9.5%), vomiting (1.6%), and reduced appetite (1.6%). The subjective impression of fatigue was supported by data from the 24 h protocols of group A showing that the proportion of sleep per 24 h increased from 10.1 (SD: 2.3) to 11.3 (SD: 2.2) hours (z = 3.952; *p* = 0.004). Daytime sleep phases (8 am–8 pm) increased from 2.5 (SD: 1.6) to 3.3 (SD: 1.9) (z = 3.306; *p* = 0.004).

The effective dose of DRB was significantly higher in patients who experienced fatigue as a side effect. In patients with fatigue, the mean DRB dose was 0.74 (SD: 0.71), whereas in patients without fatigue, it was 0.27 (SD: 0.19) mg/kg/bw/day (z = 4.100; *p* = 0.006, r = 0.346). Only one of the 14 patients with fatigue had started DRB on the unit. A similar result was found for behavioral problems, where the average DRB dose was 1.03 (SD: 0.70) and 0.25 (SD: 0.17) mg/kg/bw/day in the absence of those symptoms (z = 8.581, *p* < 0.001, r = 0.52). All nine patients with behavioral problems had started DRB as outpatients. Other medications (especially opiates and/or sedatives) were not used more frequently in relation to the two most common side effects.

In the fourteen patients with fatigue, an opioid was used in three cases (χ^2^ (2) = 1.168; *p* = 0.637) and a sedative in three other cases (χ^2^ (2) = 0.919; *p* = 0.524). Among the nine cases with behavioral abnormalities, two were treated with opiates (χ^2^ (2) = 1.291; *p* = 0.558) and three with sedatives (χ^2^ (2) = 0.902; *p* = 0.632). All side effects resolved completely.

**Figure 2:**
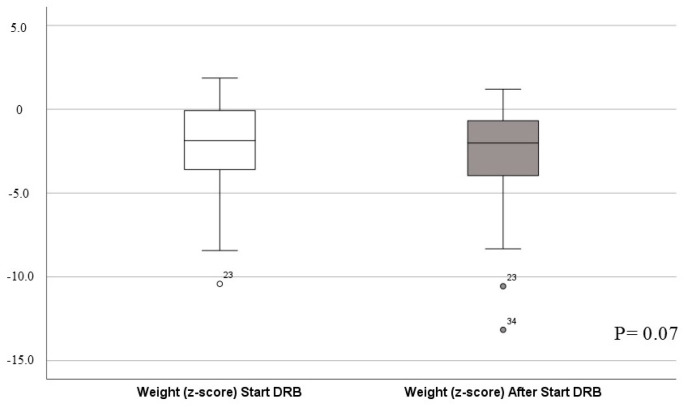
Weight differences.

Study patients weighed significantly less than the normal population at the start of DRB treatment, also losing more weight over the course of the study. Examining the number of standard deviations from the mean value of the age-specific normal weight, the patients had a z-value of −2.3 before and −3.2 after the start of DRB treatment (see Figure 2). This difference was not statistically significant.

### 3.6. Outcome

Treatment with DRB was stopped in 12 patients (=19%), due to side effects in 5% and non-efficacy in 14%, while 15.9% of the patients died in the observed period.

## 4. Discussion

The aim of the study was to investigate efficacy and safety of dronabinol (DRB) on various distressing symptoms in children with mostly neurological, life-limiting conditions. Regarding the most common symptoms, the subjective response rates were 27% for restlessness and 47.6% for spasticity. However, objective parameters showed no reduction in phases of restlessness or amounts of demand medication. Three patients experienced severe side effects, with other effects being fatigue (22.2%) and behavioral problems (14.2%).

Compared to other studies, we observed similar gender and age distributions, as well as underlying diseases [11,13,18,24]. The boy-to-girl ratio of 1.7:1 in this study is comparable to other studies involving pediatric patients with LLCs and neurological impairment [11,12,13,14,15,24,25]. In the cited studies, a total of 226 patients were included, of which 153 were male, yielding a ratio of 2.1:1. Similar differences have been found in various studies, such as those on cerebral palsy (CP), which shows a higher rate in males [26,27]. Various causal factors are being discussed that could trigger increased biological neurological vulnerability in males [28,29], but our data cannot explain this difference.

Nevertheless, we observed a highly selected group of children for whom outpatient therapy was insufficient. The patients in this study were treated exclusively on one PPC unit, and the severity of their symptoms is likely more pronounced than in the outpatient setting.

A strength of this study is the standardized observation of the patients through 24 h protocols and the reports from various professional groups, which were documented one to three times a day. However, the positive effects (good to moderate response) on distressing symptoms noted by the nursing staff and doctors, which ranged from 0–38%, could not be reproduced in the standardized patient documentation. There were no significant reductions in seizure frequency or anticonvulsant on-demand medication. The same results were observed for restlessness. While the subjective response was over 25%, the 24 h protocols showed neither a reduction in restlessness phases nor a reduction in demand sedative medication. The subjective reduction in symptoms is consistent with data from other case series or retrospective studies [11,13,24,25]. However, these studies usually lack the standardized observations of a multi-professional palliative care unit.

A prospective study by Libzon compared two different mixtures of THC and cannabidiol (CBD) in 25 children with moderate to severe impairment of motor function. Over the course of five months, significant improvements were observed in spasticity, dystonia, pain, and sleep behavior [25]. These different results could be due to the combination therapy and the lower disease severity compared to our study, which involved a higher number of medications, a larger proportion of GMFCS 5, and more feeding tubes.

A retrospective study by Tagsold with 31 patients showed a good response of up to 64.5% on various symptoms [30], which contradicts our data. In Tagsold’s study, four different medicinal cannabinoids were administered, with only 20 of the 31 patients receiving DRB. In contrast to our sample, one-third of the children had oncological diagnoses, and the evaluation of treatment success was mono-professional without systematic symptom recording.

This raises the problem that attending physicians, as in our study, identify successes that do not stand up to structured observation in the inpatient setting. The higher subjective response rate could be the result of confounding.

The narrative of medical cannabis or cannabinoids gives hope that a plant-based (and potentially well-tolerated) medicine could alleviate the distressing symptoms of severely ill children. In this context, Moore and colleagues developed a narrative bias tool for studies on medicinal cannabis [31]. The tool showed that just reading the title and abstract of a study on medicinal cannabis could lead to an incorrect impression of safety and efficacy in 20% of cases. This reinforces the need to collect more data on this topic and discuss it critically. Arguments in favor of closely monitoring the side effects of dronabinol include the three cases of potentially life-threatening events. Previous experience with respiratory problems in adult women was published in the 19th century [32]. In contrast, our patients are younger, extremely underweight, vulnerable, and treated with multiple medications as well as morphine and sedatives. The severe side effects analyzed here occurred exclusively during dosing in the first weeks. If these had occurred in the outpatient setting, they might not have been noticed early on and treated sufficiently. By coincidence, patients in group B might have experienced more frequent—but less recognized—effects. This could explain why the average dose was higher in the outpatient group. The relationship between higher dose and more side effects should be considered in a future standard for DRB treatment.

Low body weight is a problem in pediatric palliative care. For example, cachexia in children with chronic renal insufficiency is correlated with increased morbidity and an increased risk of hospitalization [33]. Other consequences of low body weight are an increased risk of developing pneumonia [34] and decubitus [35]. Cannabis may stimulate appetite and promotes weight gain [36], but experiences vary. For example, chronic cannabis use reduces appetite in psychiatric patients [37], but there is evidence of increased appetite in adult cancer patients [38]. In our study, there was a trend towards weight loss; however, this effect was not significant. Importantly, patients with severe neurological impairment may not be able to communicate hunger directly. Indirect signs of hunger may resemble other discomforts and could cause restlessness. Here, a vicious circle could emerge for our patients, which should be considered before and during therapy with THC-containing drugs.

A similar thought develops regarding the emotional side effects of DRB, which were observed in both our patients and in other studies. After oral administration of tetrahydrocannabinol (THC) and cannabidiol (CBD), healthy volunteers reported anxiety, dysphoria, and psychotic symptoms [39]. These undesirable effects may also be less easily communicated by children on a PPC unit.

In recent years, there have been more reports on the long-term effects of cannabis use, such as an increased risk of psychotic disorders [40]. In our sample, the survival rate at 5 years after the start of DRB was almost 70%. In this respect, long-term consequences of THC should be considered in pediatric palliative care.

### 4.1. Limitations

The retrospective design of this study results in a lack of dose control and errors in recording responses and side effects. For example, changes in anticonvulsants were made before and after the start of DRB, which could confound the results. The completeness of the of the patient records may have varied. The number of patients is low, especially those who started therapy on the ward. On the other hand, the number of different life-limiting conditions is large, even if the comorbidities and painful symptoms are very similar. Side effects such as fatigue cannot be reliably separated from the increasing progression of neurodegenerative diseases. However, the proportion of progressive diseases (=TsfL-3) was relatively low (under 25%).

### 4.2. Proposal for a Standard DRB-Treatment of Restlessness

**Figure 3:**
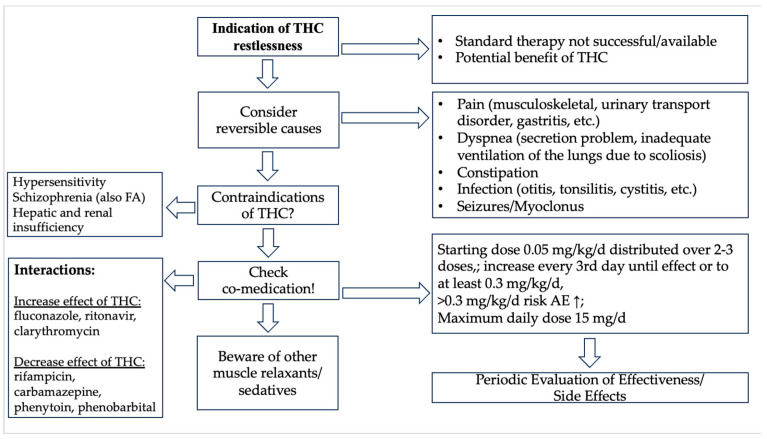
Proposal of DRB treatment for inpatients with restlessness. FA = family anamnesis; THC = tetrahydrocannabinol.

The results of this retrospective study led to the development of a simple, practicable scheme to define the indication for treatment with dronabinol for restlessness, the most common symptom. High DRB dosages > 0.5 -> 1.0 mg/kg/bw) appear to be associated with more frequent side effects. Thus, it was important to include a dosage suggestion. Another aim was to review contraindications and practical aspects. A further aim was to improve standard of care to compare future patients. Due to the potentially life-threatening side effects, treatment with DRB should be started in the inpatient setting. Details can be found in Figure 3.

## 5. Conclusions

In this retrospective study of children and adolescents with LLCs, DRB had a subjectively graded effect on typical symptoms (spasticity > restlessness > pain > seizures > dystonia > myoclonus). The symptoms were not significantly reduced according to standardized protocols or the use of demand medication. Moreover, there were patients with severe side effects. DRB can be used if standard medications have failed. Indications and contraindications must be discussed carefully. The initiation of DRB therapy should be monitored in an inpatient setting. Prospective studies are needed to investigate and verify the retrospective results. The larger outpatient area of pediatric palliative care should also be better investigated.

## Figures and Tables

**Table 1:** Characteristics of all study patients.

Patients	N	63
Gender	Female: Male:	36.5% 63.5%
Age Mean/SD Age Median		10.4 (SD 6.3) years 9.3 years
Palliative Groups:	TfSL-1:	4.8%
	TfSL-2:	1.6%
	TfSL-3:	24.2%
	TfSL-4:	69.4%
Disease Groups:	Neurologic disease: • Hypoxic: • Metabolic: • Malformation: • Epileptic syndrome: • Combination/Unclear: • Tumor: No neurologic disease:	34.9% 25.4% 23.8% 6.3% 3.2% 3.2% 3.2%
Co-Morbidity:	Psychomotor Retardation:	92.1%
	Epilepsy:	92.1%
	Cerebral Palsy:	88.9%
	Respirator Therapy:	3.2%
Co-Medication to Dronabinol:	Antiepileptic Drugs (AED): Number of AED (mean/SD): Benzodiazepines: Opiates: Skeletal Muscle Relaxants: Other Sedatives: Cannabidiol (counted as AED also):	92.1% 1.95 (SD 1.03) 28.6% 30.2% 50.8% 15.9% 4.8%

AED = antiepileptic drugs; TfSL = Together for Short Lives.

**Table 2:** Dronabinol treatment.

		Start Dronabinol in Hospital			Adjusted *p*-Value ^b^	Effect Size ^c^
		Yes (=A) N = 26	No (=B) N = 37	*p*-Value ^a^		
Indication DRB	Spasticity: Restlessness: Dystonia: Pain: Seizures: Myoclonus:	18 (69.2%) 19 (73.1%) 6 (23.1%) 11 (42.3%) 5 (19.2%) 3 (11.5%)	24 (64.9%) 26 (70.3%) 1 (2.7%) 25 (67.6%) 6 (16.2%) 3 (8.1%)	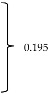	n/a	n/a
Start DRB after (A)/before admission (B)	(mean/SD): Median:	10.1/7.0 8	174/137 136	n/a	n/a	n/a
Dosage DRB (mg/kg BW/day) (mean/SD)	Starting dosage: Effective dosage: Last dosage: Dosing Steps:	0.06/0.21 0.21/0.11 0.25/0.21 3.12/1.12	n/a 0.48/0.50 0.46/0.49 n/a	n/a 0.007 0.013 n/a	n/a **0.01** **0.01** n/a	n/a 0.330 0.300 n/a
Treatment period (days)	(mean/SD): Median:	739/939 259	369/390 240	0.939	0.939	0.008
Follow-up (month)	Median: Range:	16.6 0.5–100.9	1.8 0.2–48.2	0.001	**0.004**	0.320

^a^ = Mann–Whitney U-test/Chi-square-test, ^b^ = Benjamini/Hochberg, ^c^ = Cohen, n.a. = not adjusted.

**Table 3:** Severe side effects.

Patient-Number/Age	Description	Dosage at Time of Event (mg/kgBW/day)	Co-Medications Modified	Course	Late Sequelae
22/ 13 y, 0 m	Day 3 after start of DRB: Respiratory insufficiency Oxygen-Inhalation	0.09	No	Resolution after stopping and withdrawal of DRB	no
25/ 1 y, 6 m	Day 14 after start of DRB: Respiratory insufficiency Non-invasive ventilation	0.3	No	Resolution after stopping and withdrawal of DRB	no
35/ 9 y, 6 m	Day 12 after start of DRB: Respiratory rate reduced	0.25	No	Resolution after stopping and withdrawal of DRB	no

## Data Availability

The data presented in this study are available upon request from the corresponding author. The data are not publicly available due to privacy issues.
